# The College of Nursing (Limited by Guarantee)

**Published:** 1921-02-26

**Authors:** 


### THE COLLEGE OF NURSING

**(Limited by Guarantee.)**

### What the Local Centres are Doing.

### EAST LANCASHIRE CENTRE.

(Hon. Secretary, Miss Earl, Ancoats Hospital, Manchester.)

The "Rowan" Midwifery Scholarship.

Miss Dorothy A. Cox, of the Royal Sea Bathing Hospital, Margate, has won this scholarship, which is valued at £30 and was offered by the East Lancs Local Centre for members of the College of Nursing who received their training in East Lancashire.

Next Meeting.

Wednesday, March 2, 3.30 p.m., at the Manchester Royal Infirmary.—Miss Burstall, Head Mistress of the Manchester High School for Girls, on "The Art of Public Speaking." Admission by card of membership; non-members on payment of 1s.

Lantern Slides of Florence.

The Rev. Mr. Fripp delighted his audience, on the 16th inst., with an illustrated lecture on "Giotto and his Bell Tower in Florence."

### EDINBURGH CENTRE.

(Hon. Secretary, Miss Cathcart, The Elms, Whitehouse Loan.)

Monday, February 28, 7.30 p.m.—A Social Evening, preceded by short Business Meeting, will be held in the Nurses' Club, 8 Drumsheugh Gardens. Members are asked to intimate whether they can attend. Refreshments, 1s.

### GLASGOW CENTRE.

(Hon. Secretary, Miss E. Moseley, Western District Hospital, Oakbank.)

Saturday, February 26, at 3 p.m., in the Glasgow Nurses' Club (Miss Roy Reid), 10 Claremont Terrace, W.: Meeting and a lecture on "Surgical Tuberculosis," by J. Taylor, Esq., F.R.C.S. (Edin.), Surgeon to Glasgow Corporation Hospitals for Tuberculosis. Admission: Members, free; non-members, 1s.

### LONDON CENTRE.

(Secretary, Miss M. A. Bompas, 7 Henrietta Street, W. 1.)

Nominations for the Council.

Monday, February 28, 8 p.m.—A very important General Meeting of the London Centre will be held at St. Thomas's Hospital. Members are asked to make a point of being present, as important questions will be discussed.

We are requested to state that this meeting has been specially convened to give the members an opportunity to meet the candidates nominated for the Council elections by the members at the last meeting. It is hoped that this will be a help to those members who do not personally know any of those who are able to stand for election. Although it is obvious that to obtain the support of a College Centre for any candidate is greatly to increase her chances of election, it should be remembered that every member is quite free to vote for whom she wishes. Organisation through the College Centres is merely an attempt to make known a particular candidate to a greater number of members. Every member of a Centre is privileged to nominate anyone she wishes. As there are at present at least ten candidates nominated, and only eight vacancies in England and Wales, the number to be supported officially by the London Centre must be considerably reduced if any of those nominated are to be elected. It is therefore proposed that, after having heard the candidates at the meeting on Monday next, a postal ballot shall be taken as to the four candidates the London Centre should support. The names of these four will then be circulated to all members and to the Hon. Secretaries of all the other Centres asking that these candidates may receive their support at the time of the College Council election. It is earnestly hoped that every member of the London Centre will endeavour to be present at St. Thomas's Hospital on Monday next at 8 p.m.

Forthcoming Events.

Thursday, March 10, 8 p.m.—Lecture at the Rooms of the Medical Society, 11 Chandos Street, on "The Vote, and what it has Done for Women," by Miss Eleanor Rathbone, C.C., J.P. This is the last lecture of the session, and it is hoped there will be a good attendance. All nurses, whether members of the College or not, are admitted on payment of 6d.; members of the London Centre admitted free.

The London Centre will give an "At Home" at the Club the first week in March. Music and bridge. The date will be announced next week.

### NORFOLK AND NORWICH CENTRE.

(Hon. Secretary, Miss V. A. Rutter, Albemarle House, Newmarket Road, Norwich.)

Tuesday, March 1, at 8 p.m.—General meeting of members at the Norfolk and Norwich Hospital (by kind permission), to nominate candidates for the forthcoming College Council election. The Hon. Secretary will be glad to receive names of proposed candidates who have consented to stand by Monday, February 28.

Lecture.

Thursday, March 3, at 8 p.m.—Lecture at the Norfolk and Norwich Hospital by C. Noon, Esq., F.R.C.S., on Abdominal Surgery. Members, free; non-members, 1s.

### NORTHUMBERLAND AND DURHAM CENTRE.

(Secretary, Miss M. Toyne, 17 Windsor Terrace, Jesmond, Newcastle-on-Tyne.)

The next meeting will be held on Friday, March 18, at 6.30 p.m., at the Nurses' Club, 17 Windsor Terrace. It is hoped all members will endeavour to be present, as matters of great importance in connection with the College will be discussed.

On Friday, February 18, Mr. A. E. Morison gave an excellent and most interesting lecture on "Some General Principles in Orthopædic Surgery," which was much enjoyed.

### YORKSHIRE CENTRE, LEEDS.

(Hon. Secretary, Miss Lindall, Hospital for Women and Children.)

Nomination for Council Election.

Friday, March 4, at 7 p.m.—Special meeting of members in the Outlook Club, Greek Street, Leeds, to nominate a candidate for the coming Council election of the College. It is requested that members representing all branches of nursing will be present. The candidate chosen should be able to attend the Council meeting in London fortnightly, for which reasonable expenses are paid by the College.

Miss MacGregor's Address.

On Friday, February 11, a most interesting address was given by Miss E. Sheriff MacGregor, in the Clinical Theatre of the Leeds General Infirmary, on "Roumania and Russia in War-time." The vivid account of the different countries and towns visited on the journey to nurse in Roumania was given in a most able manner, and much enjoyed by all present.

